# Fabrication of Glass Fiber Reinforced Composites Based on Bio-Oil Phenol Formaldehyde Resin

**DOI:** 10.3390/ma9110886

**Published:** 2016-11-01

**Authors:** Yong Cui, Jianmin Chang, Wenliang Wang

**Affiliations:** 1 College of Materials Science and Technology, Beijing Forestry University, Beijing 100083, China; 00767@siit.edu.cn (Y.C.); wangwenliang@sust.edu.cn (W.W.); 2 Precision Manufacturing Engineering Department, Suzhou Vocational Institute of Industrial Technology, Suzhou 215104, China; 3 College of Bioresources Chemical and Materials Engineering, Shanxi University of Science & Technology, Xi’an 710021, China

**Keywords:** bio-oil, phenol formaldehyde resin, glass fiber reinforced, composites

## Abstract

In this study, bio-oil from fast pyrolysis of renewable biomass was added by the mass of phenol to synthesize bio-oil phenol formaldehyde (BPF) resins, which were used to fabricate glass fiber (GF) reinforced BPF resin (GF/BPF) composites. The properties of the BPF resin and the GF/BPF composites prepared were tested. The functional groups and thermal property of BPF resin were thoroughly investigated by Fourier transform infrared (FTIR) spectra and dynamic thermomechanical analysis (DMA). Results indicated that the addition of 20% bio-oil exhibited favorable adaptability for enhancing the stiffness and heat resistance of phenol formaldehyde (PF) resin. Besides, high-performance GF/BPF composites could be successfully prepared with the BPF resin based on hand lay-up process. The interface characteristics of GF/BPF composites were determined by the analysis of dynamic wettability (DW) and scanning electron microscopy (SEM). It exhibited that GF could be well wetted and embedded in the BPF resin with the bio-oil addition of 20%.

## 1. Introduction

As an important alternative of thermoplastic engineering materials, glass fiber (GF) reinforced phenol formaldehyde resin (GF/PF) composites has many desirable properties for application, such as low specific mass, high rigidity, good corrosion resistance, excellent flame retardation, and less smoke and toxicity when burning [1,2].

However, the environmental stress and price fluctuation caused by petroleum-based phenol obtained from fossil energy has severely limited the application of GF/PF composites. Besides that, drawbacks of traditional phenol formaldehyde (PF) resin, such as high brittleness, insufficient thermostability and plenty of free phenol and formaldehyde [3,4], have meant that GF/PF composites cannot be used in aerospace, transportation, interior and other fields, where the performance of materials needed to be upgraded. In order to extend the application range of GF/PF composites, the study of modifying PF resin has increased dramatically during last few years [5,6,7,8,9,10,11].

Bio-oil obtained from fast pyrolysis of renewable biomass has abundant amounts of phenols, aldehydes, ketones, and hydrocarbon [12,13,14], and can be used for preparation and modification of PF as an environment-friendly and cost-saving raw material. In recent years, extensive studies have been conducted concerning the synthesis of bio-oil phenol formaldehyde (BPF) resin. Chum et al. [15] utilized fast pyrolysis of pine sawdust to produce bio-oil and separated a phenolic-rich fraction from the raw oils by water and aqueous sodium bicarbonate washing. After removing acids, a novolak formulation with 50% of phenol and 50% of the phenolic-rich fraction was successfully prepared. Kelley et al. [16] used the phenolic-rich component extracted from bio-oil as a low-cost replacement for petroleum-derived phenol in the synthesis of PF resin. This work found that when 25 wt % of the phenol was replaced with the phenolic-rich component, the performance properties of properly formulated BPF resin compared favorably with those of commercial PF resin. Chan et al. [17] replace 25 wt % and 35 wt % of phenol in PF resin with phenolic-rich oil derived from softwood bark residues vacuum pyrolysis and developed the new type of BPF resin for wood adhesive. It was concluded with the performance of BPF resin, that bio-oil can replace up 35 wt % phenol in PF resin for OSB manufacturing. Aslan et al. [18] found that the phenol-rich fraction of crude bio-oil could partially substitute for the petroleum-based phenol in commercial PF resin adhesives and there was a similar molecular structure between BPF and PF resin. Lee et al. [19] prepared alcohol-soluble PF resin with the raw material of bio-oil and phenol mixed in weight ratios of 50/50, 40/60, and 30/70 and investigated the molecular weight distribution, thermal setting behavior and heat resistance of BPF resins. The results showed that bio-oil blended with phenol was a suitable raw material for preparing alcohol-soluble PF resin. Amen-Chen et al. [20,21,22] synthesized BPF resin for OSB adhesive, with different levels of phenol replacement by phenolic-rich bio-oils, and evaluated mechanical and physical properties of OSB. Three-layer panels, prepared with resins having 50 wt % phenol replacement in the surface and 25 wt % phenol replacement in the core, had mechanical properties above the requirements specified by Canadian Standards.

All the earlier literature indicated that using bio-oil in the synthesis of PF resin is feasible, having a lower cost and toxicity [23]. Although applied in wood adhesive, wood modifier [24], activated carbon [25], previous BPF resins were not suitable to fabricate glass fiber reinforced composites, due to low solid content, high viscosity and high curing temperature. Furthermore, as the relatively low activity of bio-oil, the adhesive properties of BPF resin synthesized with the method of phenol replacement by bio-oil was weaker than that of commercial PF resin.

In the present work, bio-oil was added to synthesize specific BPF resins while the content of phenol and the mole ratio were remained consistently, which could help maintain the inherent structure of PF resin. Then, the specific BPF resin was used to fabricate GF reinforced BPF resin (GF/BPF) composites, aiming to improve the performance of PF resin based materials. The performance of BPF resins and prepared GF/BPF composites were characterized using corresponding standards. The chemical structure and thermodynamic properties of BPF resins were characterized by Fourier transform infrared spectroscopy (FTIR) and dynamic thermomechanical analysis (DMA). The interfacial properties of GF/BPF composites were characterized by dynamic wettability (DW) and scanning electron microscopy (SEM).

## 2. Experimental

### 2.1. Materials

The bio-oil from fast pyrolysis of larch sawdust was supplied by the Institute of Wood Based Material (Beijing Forestry University, Beijing, China). The bio-oil contained 22% phenolic, 24% ketone, 20% aldehydes, 16% organic acids, 8% sugars, 4% hydrocarbons and other compounds, and had been adjusted to the pH value to 7.1–7.3. The GF cloth with the thickness of 0.6 mm contained 0%–2% metallic oxide, provided by Weisheng Composites Company (Wuxi, China). The phenol, paraformaldehyde, sodium hydroxide, silane (KH-550), and acid were reagent grade and used as obtained without purification.

### 2.2. Preparation of BPF Resin

To effectively reflect the concerned information when bio-oil was used in BPF resin synthesis, BPF resins were prepared by batch copolymerization with bio-oil adding amounts of 0 wt %, 10 wt %, 20 wt %, 30 wt % and 40 wt % (in relation to the mass of phenol), designated as PF, 10%-BPF, 20%-BPF, 30%-BPF and 40%-BPF, respectively. To get the resins of high solid content and low viscosity without a distillation process, paraformaldehyde was used to replace formaldehyde solution (37 wt %). The mole ratio of phenol to formaldehyde was set as 1:1.8 and the mole ratio of phenol to NaOH was fixed at 1:0.25 in this study. The specific steps of conducting the BPF resin samples were as follows:

1. At first, phenol and 70% of the total NaOH solution (30 wt %) were mixed in a three-necked flask. The mixture was heated to 70 °C in 30 min.
2. Then, 80% of the total paraformaldehyde was added in batches, avoiding boiling. The mixture was kept at 70 °C–75 °C until completely depolymerized.
3. Next, the remaining 30% of the total NaOH solution (30 wt %) was added. The mixture was heated to 80 °C in 30 min and then kept for 15 min.
4. After the temperature was dropped to 70 °C, the remaining 20% of the total paraformaldehyde and bio-oil was added in batches.
5. Finally, the mixture was heated to 85 °C–90 °C and held for 45 min. When the reaction was complete, it was rapidly cooled to 40 °C to yield the BPF resin.

In addition, BPF resin castings with the size of 15 × 6.5 × 3 mm³ (length × width × thickness) were separately prepared to meet the test of heat resistance, using acidic curing agent at ordinary temperature.

### 2.3. Preparation of GF/BPF Composites

The GF/BPF composites were manufactured using a hand lay-up process at ordinary temperature. The GF/BPF composites with the size of 150 × 150 mm^2^ were fabricated at a target thickness of 4 mm, adding acidic curing agent content of 5% and silane coupling agent (KH-550) content of 0.5% by the weight of BPF resin. The acidic curing agent consisted of p-toluenesulphonic acid, sulfuric acid, phosphoric acid and ethanol with mass ratio of 1:0.1:1:0.3. GF/BPF composites were made from BPF resins with different amount of bio-oil adding, resulting in a total of five types of panels. The process method was conducted in the preparation of GF/BPF composites as shown in the following steps:

1. The GF cloth with the size of 150 × 150 × 0.6 mm^3^ was immersed in ethanol solution for half an hour and then dried at 60 °C for 2 h.
2. After surface clearing and drying, the mold which was made of acrylic resin in the size of 200 × 200 × 1 mm^3^, was coated with solid paraffin as the release agent.
3. Next, the exact amount of acidic curing agent and silane coupling agent were gradually added to BPF resin and the mixture resin was blended for 10 min.
4. Then, one piece of treated GF cloth was laid on the mold and coated with the same weight of mixture resin. Next, another piece of cloth was laid on the previous one and coated with mixture resin. The above operation was repeated until the composites achieved the ideal thickness, and held for 24 h at 25 °C to cure and mold. Usually, 6 pieces of GF cloth were needed to achieve the ideal thickness and the dial thickness gauges were used to measure the thickness.
5. After demolding and further curing at 70 °C for 2 h, the composites was finally prepared.

### 2.4. Characterization of BPF and GF/BPF Composites

The viscosity, solid content, gel time, free formaldehyde and free phenol of BPF resins were determined in accordance with the Chinese National Standard (GB/T 14704-2006, GB/T8924-2005). The FTIR (Vertex 70, Bruker, Karlsruhe, Germany) was used to characterize the functional groups of BPF resins. The FTIF spectra of BPF resins ranged from 4000 cm^−1^ to 400 cm^−1^. The DMA (Q800, AT Company, New Castle, DE, USA) was applied in thermodynamic analysis. Scans were run at heating rates of 3 °C min^−1^, a frequency of 1 Hz and an amplitude of 15 μm. The scanning temperature ranged from 50 °C to 400 °C. Specimens in the size of 35 × 10 × 3 mm^3^ were made from BPF resin castings before analysis. 

Bending strength and oxygen index of GF/BPF composites were measured according to Chinese National Standard (GB/T 1449-2005, GB/T8924-2005). The interfacial wettability properties between BPF resin and GF were tested by DCA (DCAT21, Data Physics Corporation, Regensburg, Germany) using the GF of 0.03 mm diameter. The testing angle ranged from 0° to 180° and the immerged depth of fiber was 3 mm at the speed of 1 mm/s. The SEM (Quanta 200, FEI Company, Hillsboro, OR, USA) was used to characterize the fracture surface parallel to the thickness direction. The fractured surfaces of the specimens were frozen in liquid nitrogen for 5 min and sputter-coated with gold powder using a SCD-005 sputter coater.

## 3. Results and Discussion

### 3.1. Characterization of BPF Resins and GF/BPF Composites

The prepared BPF resins using different additions of bio-oil were all dark-brown aqueous solutions, containing little water compared to other PF resins that were made of formaldehyde solution (37 wt %). Table 1 shows the viscosity, solid content, gel time, free formaldehyde and free phenol content of BPF resins. The oxygen index and bending strength of GF/BPF composites were also characterized. Although the structure of natural polyphenol was similar to petroleum phenol, the complex multi-component system of bio-oil made the performance of BPF resins significantly different from commercial PF resin. In spite of applying the same synthesis process, considerable difference in properties was also found among the BPF resins with various addition of bio-oil.

The viscosity of BPF resins were lower than that of PF resin and gradually reduced with the bio-oil addition increasing. This happened because the prepared PF resin in this study may be diluted by the water and other organic compounds in bio-oil, which did not participate in the synthesis process. It is known that a too high or low viscosity will bring adverse effects on fabricating GF/BPF composites by hand lay-up process. The result showed that the viscosity of PF resins could be adjusted to a right level using bio-oil. When the adding amount of bio-oil was 20%–30%, the viscosity was from 590 mPa·s to 730 mPa·s, which was considered to be suitable in the application.

The solid content of BPF resins was more than 70%, which was obtained without water distilling by using paraformaldehyde, but lower than that of PF resin. High solid content was beneficial to form a continuous and stable bond line in the final products. For most resins, viscosity and solid content were closely related to each other, therefore the solid content of BPF resins also showed the similarly reduced trend with bio-oil addition increasing.

The gel time of BPF resin was continuously extending with bio-oil addition increasing, when the same curing process was applied. It was generally accepted that bio-oil was less reactive than petroleum phenol and notable negative effects would be brought to the inherent curing process of PF resin if too much bio-oil were added [26]. According to the experience of preparing GF/BPF composites, the appreciated gel time was from 22 min to 25 min. In that case, operation time and production efficiency could be simultaneously satisfied.

The free formaldehyde content of BPF resin was less than that of PF resin, and that trend became more evident with the bio-oil addition increasing. This implied that natural polyphenol in bio-oil was able to cause a hydroxyl-methylated reaction with formaldehyde during the synthesis process, resulting that only a slight amount of free formaldehyde remained in BPF resin.

The content of free phenol in all BPF resins was higher than that of PF resin. This may be due to the fact that the amount of natural polyphenol obtained from bio-oil addition was continuously increasing, while most formaldehyde in the system was consumed by the synthesis reaction. In order to reach the standard of low-toxicity resin, the content of free formaldehyde and free phenol must be below the limited requirement at the same time. Depending on the result in Table 1, when the bio-oil addition was within the scope of 10%–20%, the properties of BPF resin could be regarded as environment-friendly.

With an increase in bio-oil addition from 0% to 40%, the main properties of GF/BPF composites first increased and then decreased. It was found that the oxygen index, bending strength and modulus of elasticity (MOE) reached their maximum values at the bio-oil addition of 20%, which indicated that bio-oil was beneficial to modify the brittleness and thermostability of PF resin composites. However, when the bio-oil addition rose beyond the limit of 20%, the less reactive of bio-oil regarded as the inherent drawback would become clear, especially in a high addition. In addition, the other properties of bio-oil, such as the water and ash content, may also affect the performance of BPF resins and composites. Thus, the performance of GF/BPF composites prepared using a high bio-oil addition was lower than that made from a low bio-oil addition.

The actual result showed that bio-oil could fully react with the formaldehyde-phenol system and had perfect feasibility for modifying the performance of GF/PF composites. The BPF resin synthesized with the suitable proportions of bio-oil addition exhibited some advantages, such as satisfying performance in preparation as well as being low-cost and low-toxic. Furthermore, the good stiffness and heat resistance would further extend the scope of the applications of GF/BPF composites. The overall performance of the BPF resin synthesized with the suitable bio-oil addition was better than that of PF resin in preparing GF reinforced composites.

### 3.2. FTIR Spectrum Analysis

**Figure 1:**
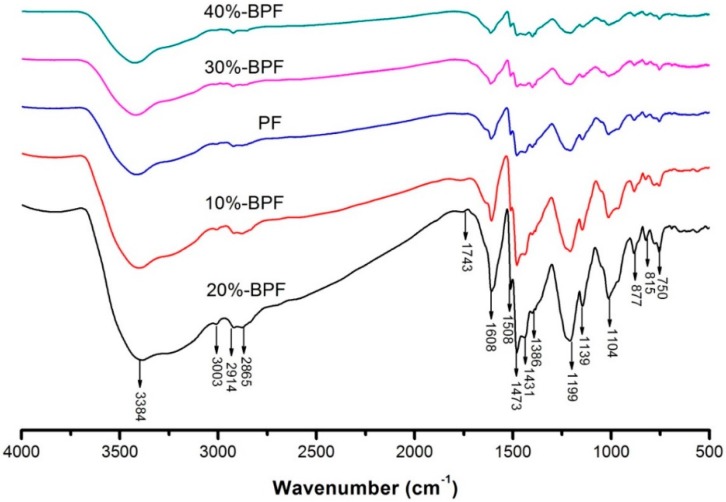
FTIR spectrum of various BPF resins.

To investigate the chemical structure of BPF resin synthesized with various amounts of bio-oil addition, the 4000–400 cm^−1^ infrared spectral region was shown in Figure 1. The PF resin was also analyzed as a comparison. Following recognized peak identifications of the infrared spectra of organic compounds, functional groups corresponding to the major peaks in the spectra were identified and listed in Table 2. The BPF resins had FTIR spectra similar to that of PF resin, suggesting that the BPF resins exhibited similar molecular structure as compared to the PF resin. 

A broad peak at 3384 cm^−1^ was assigned to the stretching vibration of hydroxyl and a weak band at 460 cm^−1^ was attributed to the out-of-plane deformation mode of hydroxyl [27]. Besides, an accompanied band around 1199 cm^−1^ and 1104 cm^−1^ was separately assigned to the stretching vibration of phenol C–O and benzyl hydroxyl C–O [28]. These peaks could be used to indicate the existence of phenolic hydroxyl and hydroxyl methyl in the resin. As shown in Figure 1, the peaks attributed to phenolic hydroxyl and hydroxyl methyl were remarkable in the 20%-BPF resin. It was known that methylene bridges were mainly formed by a dehydration condensation reaction between hydroxyl methyl and the unreacted active hydrogen atoms in benzene ring [29]. Therefore, the crosslinking structure could be formed easily in the 20%-BPF resin. 

The weak peak at 3003 cm^−1^ was associated with the stretching vibration of CH in the benzene ring. The peak at 1608 cm^−1^ as well as two remarkable shoulder peaks at 1508 cm^−1^ and 1473 cm^−1^ were ascribed to stretching vibration of C=C in benzene rings. These peaks were able to prove the existence of benzene rings. A group of bands could be observed at 877 cm^−1^, 815 cm^−1^ and 750 cm^−1^, where the bending vibration of C-H in the aromatic rings was characteristic [30]. The peaks related to above groups were sharp in the 20%-BPF resin.

The peaks at 2914 cm^−1^ and 2865 cm^−1^ were associated to the symmetric and asymmetric stretching vibration of aliphatic CH [29]. The bands at 1431 cm^−1^ and 1386 cm^−1^ corresponded to the bending vibration of aliphatic -CH_2_ and -CH_3_, respectively. These peaks indicated the existence of chains of hydrocarbons, which supported modifying the brittleness of PF resin. In the 20%-BPF resin, the peaks related to methylene and methyl groups were strong. The FTIR spectra of 20%-BPF resin showed a weak peak at 1743 cm^−1^ due to the stretching vibration of carbonyl groups [31]. 

To summarize the information above, the 20%-BPF resin included lots of hydroxyl methyl and hydrocarbons, which was beneficial to form the crosslinking structure and improve the stiffness. However, when the bio-oil addition was too high (over 20%), the peaks attributed to functional groups or organic compounds were weak, as shown in Figure 1. It suggested that the inherent molecular structure of PF resin would be severely impacted if too much bio-oil existed in the synthesis system.

### 3.3. DMA Analysis

**Figure 2:**
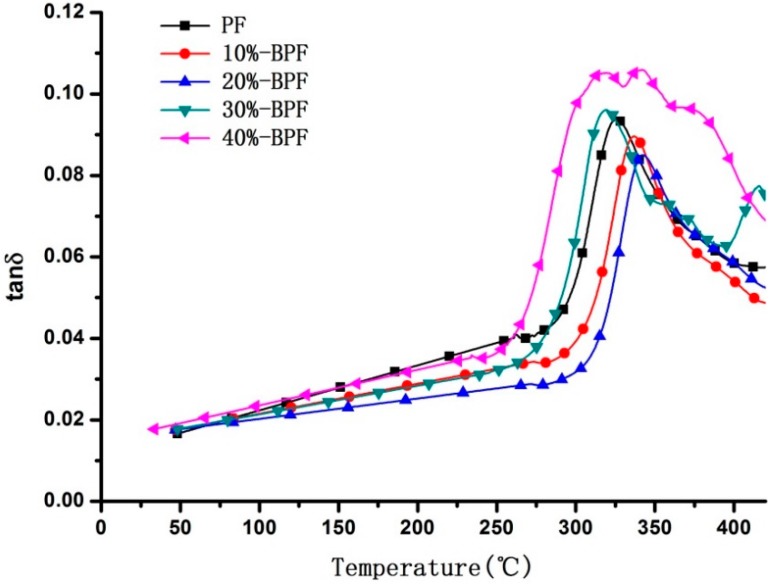
tanδ curves of various BPF resins.

**Figure 3:**
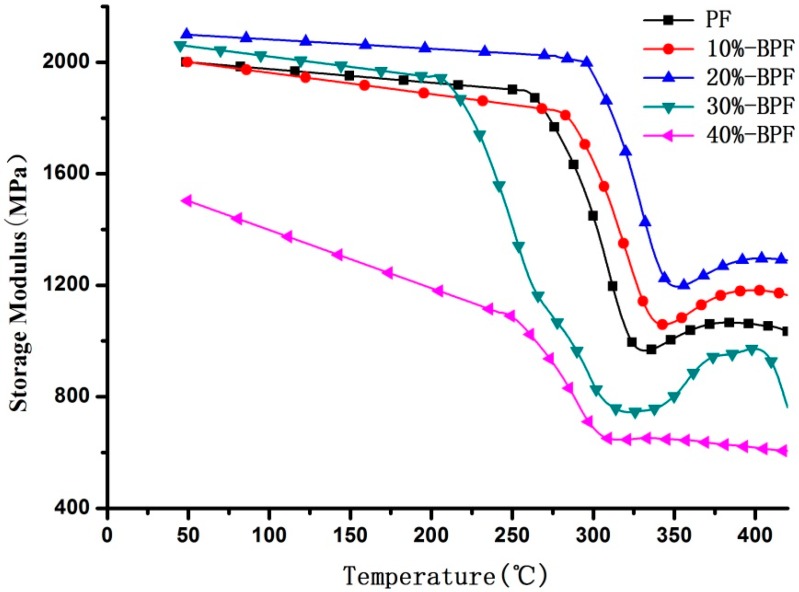
Storage modulus curves of various BPF resins.

BPF resin prepared by the reacting among phenol, formaldehyde and bio-oil under weak alkaline condition was one of resol-type resin that had thermodynamic properties. The dynamic thermomechanical analysis (DMA) of various BPF resins was summarized in Figure 2 and Figure 3. Used as various types of structural parts and components, glass-transition temperature (*T*g) was usually defined as the highest working temperatures of resin materials, below which a better combination of mechanical properties and dimensional stability could be fully satisfied. The precise information derived from the DMA demonstrated that *T*g of BPF resins first increased and then decreased with bio-oil addition increasing, and reached maximum values at bio-oil addition of 20%.

In Figure 2 and Figure 3, which showed the differential tanδ and storage modulus curves of BPF resins, 20%-BPF resin has a minimum peak value in tanδ and maximum value in storage modulus while the glass transition is occurring. It was concluded that suitable proportion of bio-oil added to PF resin would be conducive to form a three-dimensional structure in cross-linking reaction, which could improve the heat resistance of resin. However, the curve of 40%-BPF showed the maximum peak value in tanδ and minimum value in storage modulus, compared with other types of BPF resins. It was probably that excess bio-oil could not take part in the synthesis process of resin due to insufficient formaldehyde, as mentioned in the previous discussion. This moiety of bio-oil would affect the completeness of crosslinking in the resin, which led to being easily degraded and gasified as heated. Moreover, the tanδ curve of 40%-BPF resin had two obvious peaks, revealed that the molecular weight distribution was rather complicated due to excessive bio-oil existing.

### 3.4. DW Analysis

**Figure 4:**
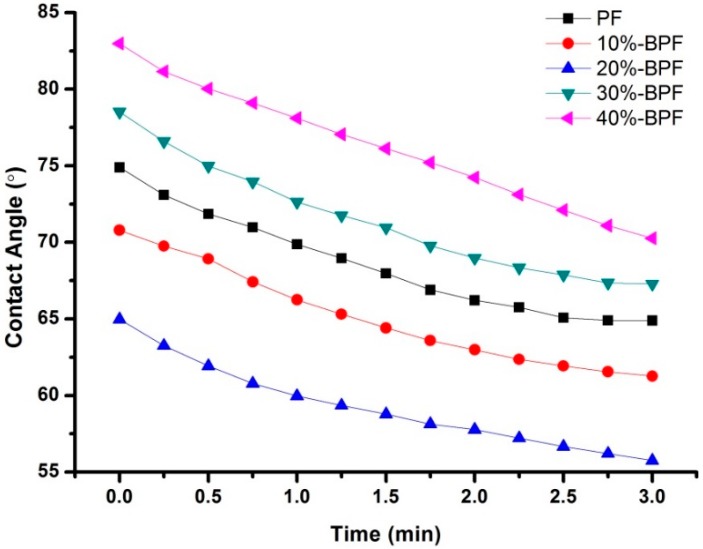
Contact angle curves of various BPF resins and GF.

The bonding results determined the final performances of GF/BPF composites; therefore it was important to investigate the interface characteristics of BPF resins and GF. As shown in Figure 4, the dynamic contact angle between various BPF resins and GF was measured. This indicated that the contact angle diminished gradually and kept steady in the end when contact time was prolonged. The contact angles were relatively low at the bio-oil addition of 20%, possibly due to including more functional groups in the 20%-BPF resin, but when the bio-oil addition was kept increasing (over 20%), bonding properties became weakened as the contact angle increased. The result of the test was consistent with the performances of GF/BPF composites listed in Table 1, implying that adding the right amount of bio-oil could improve the interface characteristics of GF/BPF composites.

### 3.5. SEM Analysis

**Figure 5:**
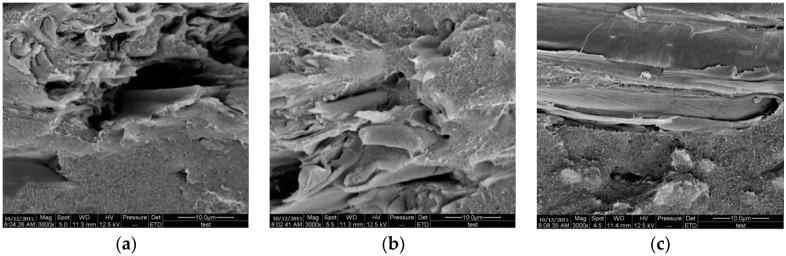
The scanning electron microscope (SEM) photograph of (**a**) GF/PF composites; (**b**) GF/20%-BPF composites; (**c**) GF/40%-BPF composites.

The SEM images of the cryogenically fractured surface of GF/BPF composites with bio-oil addition of 0%, 20%, and 40% respectively, were shown in Figure 5a–c. In Figure 5a, the fracture surfaces of GF/PF composites appeared to be smooth and flat, indicating cured PF resin with greater brittleness, and some separations and holes can be observed because of the weak adhesion between resin and fiber. When bio-oil addition reached to 20%, the fracture surface of the samples (Figure 5b) became rough and gradually transformed to be a hummocky fracture, which suggested that GF embedded in 20%-BPF resin hindered the flat extension of the fracture surface. However, when the amount of bio-oil increased to 40% (Figure 5c), the phenomenon related to the fiber pulling was evident, demonstrating that little bonding strength existed at the interface of GF and 40%-BPF resin. The above results suggested that GF could be well wetted and embedded in 20%-BPF resin, which coincided with the DW analysis.

## 4. Conclusions

Bio-oil from fast pyrolysis of renewable biomass was added by the mass of phenol to synthesize BPF resins. The results of properties testing, FTIR spectra and DMA demonstrated that the addition of 20% bio-oil exhibited favorable adaptability for enhancing the stiffness and heat resistance of PF resin. High-performance GF/BPF composites could be successfully prepared with 20%-BPF resin based on a hand lay-up process. The analysis of DW and SEM provided a valuable reference that the interface characteristics of GF/BPF composites could be improved by adding bio-oil at 20%.

## Figures and Tables

**Table 1:** Properties and glass fiber bio-oil phenol formaldehyde (GF/BPF) composites performances of BPF resin.

Resin	Resin Properties					Composites Performances		
	Viscosiy (mPa·s)	Solid Content (%)	Gel Time (min)	Free Formaldehyde (%)	Free Phenol (%)	Oxygen Index (%)	Bending Strength (MPa)	MOE ^1^ (MPa)
PF	920	79.1	16	1.8	2.8	68.2	100.2	3367.8
10%-BPF	850	77.8	22	1.3	3.1	85.6	121.4	4080.4
20%-BPF	730	76.5	25	1.1	3.5	90.2	135.5	4554.3
30%-BPF	590	74.2	29	0.9	3.8	73.5	92.5	3109.2
40%-BPF	450	72.1	32	0.85	4.6	60.4	81.6	2742.7

^1^ Modulus of elasticity was designated as modulus of elasticity (MOE).

**Table 2:** Peak identification of the FTIR spectra of BPF resins.

Wavenumber (cm^−1^)	Peak Assignment
3650–3200	OH stretch
3100–3000	Aromatic CH stretch
3000–2850	Aliphatic CH stretch
1960–1680	C=O stretch
1650–1430	Benzene ring stretch
1450 ± 10, 1375 ± 5	Aliphatic CH_3_ bend
1465 ± 20	Aliphatic CH_2_ bend
1300–1000	C–O stretch
910–650	Aromatic CH bend
